# Establishment and evaluation of a risk prediction model for coronary heart disease in primary Sjögren’s syndrome based on peripheral blood IL-6 and Treg percentages

**DOI:** 10.3389/fimmu.2024.1440370

**Published:** 2024-11-27

**Authors:** Xiaoyang Wang, Lei Huang, Bin Hu, Bin Yang, Ruipeng Wei, Shuling Rong, Bao Li

**Affiliations:** Department of Cardiology, Second Hospital of Shanxi Medical University, Taiyuan, Shanxi, China

**Keywords:** primary Sjögren’s syndrome, coronary heart disease, nomogram, erythrocyte sedimentation rate, triglycerides, interleukin-6, percentage of peripheral blood regulatory T cells

## Abstract

**Objective:**

This study aims to establish and evaluate a risk prediction model for coronary heart disease (CHD) in patients with primary Sjögren’s syndrome (pSS) based on peripheral blood levels of interleukin-6 (IL-6) and the percentage of regulatory T cells (Treg%). This model is intended to facilitate the timely identification of high-risk patients and the implementation of preventive measures.

**Methods:**

Clinical data were collected from 120 pSS patients who visited the Second Hospital of Shanxi Medical University between November 2021 and September 2023. Patients were classified into pSS and pSS-CHD groups according to CHD diagnostic criteria. Peripheral blood lymphocyte subsets and cytokine levels were assessed using flow cytometry. Univariate and multivariate logistic regression analyses were employed to identify independent risk factors, and a nomogram was constructed based on these factors. The model’s discriminatory ability, calibration, and clinical utility were evaluated using receiver operating characteristic (ROC) curves, calibration curves, and decision curve analysis.

**Results:**

The univariate and multivariate logistic regression analyses identified several independent risk factors for CHD in pSS patients: erythrocyte sedimentation rate (ESR) (OR=1.10, P=0.019), triglycerides (TG) (OR=3.67, P=0.041), IL-6 (OR=1.29, P=0.048), and Treg% (OR=0.25, P=0.004). A nomogram incorporating these factors demonstrated an area under the curve (AUC) of 0.96, indicating excellent predictive performance, and showed good calibration (P=0.599), suggesting significant clinical applicability. Furthermore, Treg% exhibited a negative correlation with cholesterol (CHOL) and low-density lipoprotein cholesterol (LDL-C) levels, while IL-6 showed a positive correlation with CHOL and LDL-C levels. TG was positively correlated with C-reactive protein (CRP).

**Conclusion:**

This study successfully developed a risk prediction model based on peripheral blood IL-6 and Treg% levels, providing critical evidence for the early identification and personalized prevention of CHD in pSS patients, with potential clinical implications.

## 1 Introduction

Primary Sjögren’s syndrome (pSS) is a common systemic autoimmune rheumatic disease (SARD) characterized by damage to exocrine glands and systemic low-grade inflammation. In China, the prevalence of pSS ranges from 0.29% to 0.77%, primarily affecting women, with a male-to-female ratio of approximately 1:9 to 1:20 (1, 2). According to the American-European Consensus Group (AECG) criteria, the prevalence of pSS in non-European populations is 2.1 to 2.3 times higher than that in European populations (3). Recent studies have indicated that patients with pSS have an increased risk of cardiovascular disease (CVD) compared to the general population (4–7). In recent years, CVD has been confirmed as a leading cause of mortality (8–10). While lymphoma was previously considered the primary cause of death, recent research suggests that pSS may be an independent risk factor for coronary heart disease (CHD) (7, 11). Therefore, predicting the risk of cardiovascular events in pSS patients is crucial for timely preventive measures.

CHD is a condition resulting from atherosclerosis caused by multiple risk factors, leading to myocardial ischemia and tissue damage. It is the most prevalent form of cardiovascular disease and a leading cause of mortality worldwide. Although traditional risk factors such as hypertension, hyperlipidemia, and diabetes are more prevalent in patients with pSS (7, 12–14), these factors do not fully account for the higher observed risk of CHD in this population. Even after adjusting for traditional risk factors, the incidence of CHD remains elevated in pSS patients (7). Therefore, the predictive factors for coronary heart disease in pSS have yet to be fully elucidated.

Inflammatory responses and immune dysregulation are hallmark features of pSS and are critical factors influencing the risk of CHD (15–18). Abnormal levels of immune cells and cytokines may contribute to the pathogenesis of pSS and are closely associated with cardiovascular risk (19–22). While inflammation and immune markers have been linked to an increased risk of cardiovascular disease in pSS patients, these factors have not been incorporated into most risk assessment tools. Existing tools, such as the Framingham risk score (23), fail to adequately account for the burden of chronic autoimmune disease-related inflammation and its impact on atherosclerosis. Although modified risk assessment algorithms have been developed for conditions like systemic lupus erythematosus and rheumatoid arthritis (24), no predictive models for the risk of CHD in pSS patients have been reported. Therefore, the development of a CHD risk prediction model tailored to pSS patients is essential.

A comprehensive analysis of various biomarkers, rather than isolated assessments, is sufficient to identify personalized prevention strategies suitable for patients. The nomogram, a visual tool for disease prediction, is widely used in cancer prognosis and clinical medicine due to its intuitiveness and accuracy (25). Recent studies have employed nomograms to assess the risk of hypertension in pSS patients and to predict cardiovascular events in patients undergoing peritoneal dialysis (26–28). Therefore, this study aims to analyze the clinical characteristics and immunological differences in pSS patients with CHD and to develop and validate a nomogram model. This model integrates data on serum lipid levels, inflammatory markers, immune cell subsets, and cytokine levels to provide a personalized prediction of CHD risk in pSS patients, thereby supporting clinical decision-making. By comprehensively analyzing biomarkers, this research will lay the foundation for developing effective preventive measures for patients.

## 2 Materials and methods

### 2.1 Clinical data

This study collected data from 120 patients diagnosed with pSS admitted to the Rheumatology or Cardiovascular Department of the Second Hospital of Shanxi Medical University from November 2021 to September 2023. In addition, there were 61 male and 59 female patients, with a mean age of 59.41 ± 10.65 years. All included pSS patients met the criteria of either the 2002 European-American Consensus Group standards (29) or the 2016 American College of Rheumatology/European League Against Rheumatism SS classification criteria (30). The diagnosis of CHD in pSS-CHD patients was based on the 2016 American Heart Association guidelines (31): patients with ≥ 50% luminal stenosis in the main coronary artery or its major branches as indicated by coronary angiography were diagnosed with CHD. Hypertension and diabetes were obtained from patient medical records and were referenced according to the “Guidelines for Prevention and Treatment of Hypertension in China (2018 Revised Edition)” (32) and the “Standards of Medical Care in Diabetes-2019” (33). Clinical and laboratory data, including blood cell counts, erythrocyte sedimentation rate (ESR), C-reactive protein (CRP), liver enzymes, bilirubin, lipid profile, immunoglobulins, numbers and percentages of peripheral blood lymphocyte subsets, and cytokines, were retrospectively collected for all patients. Blood samples were collected on an empty stomach on the day of admission. All 120 selected patients with pSS underwent coronary angiography. Based on the results, patients were divided into two groups: the pSS group (pSS) and the pSS with CHD group (pSS-CHD). This study was approved by the Ethics Committee of our institution (2024YX019).

### 2.2 Detection of the absolute and relative counts of peripheral blood lymphocyte subsets by using flow cytometry

All patients underwent venous blood collection by using heparin anticoagulant tubes. Peripheral blood mononuclear cells were isolated and prepared into appropriate concentration suspensions using density gradient centrifugation (Ficoll-Hypaque method). Subsequently, corresponding fluorescent-labeled monoclonal antibodies against human antigens were sequentially added to stain peripheral blood lymphocyte subsets. Specifically, anti-CD3-FITC, anti-CD8-PE, anti-CD45-PercP, and anti-CD4-APC were used for staining T lymphocytes; anti-CD3-FITC, anti-CD16+CD56-PE, anti-CD45-PercP, and anti-CD19-APC were used for staining B lymphocytes and NK cells; anti-CD4-FITC, anti-IFN-γ-APC were used for staining Th1 cells; anti-CD4-FITC, anti-IL-4-PE were used for staining Th2 cells; anti-CD4-FITC, anti-IL-17A-PE were used for staining Th17 cells; and anti-CD4-FITC, anti-CD25-APC, anti-FOXP3-PE were used for staining Treg cells. The fluorescent-labeled monoclonal antibodies used in this experiment were purchased from BD Biosciences and experiments were conducted according to the manufacturer’s instructions. Absolute and relative counts of peripheral blood lymphocyte subsets were determined using the FACSCalibur flow cytometer and BD Multitest software (BD Biosciences, Franklin Lakes, NJ, USA).

### 2.3 Detection of cytokine levels by using flow cytometry

Serum was obtained by centrifugation within 1 hour of blood collection and stored at -20°C. Flow cytometry was employed to measure the levels of seven cytokines in the serum, including interleukin-2 (IL-2), interleukin-4 (IL-4), interleukin-6 (IL-6), interleukin-10 (IL-10), interleukin-17 (IL-17), interferon-gamma (IFN-γ), and tumor necrosis factor-alpha (TNF-α). The CBA assay kit used was purchased from Jiangxi Cellgene Biotechnology Co., Ltd. (Jiangxi, China), and experiments were conducted according to the manufacturer’s instructions.

### 2.4 Statistical analysis

This study utilized SPSS 27.0 software (SPSS Inc., Chicago, IL, USA) and R software (version 4.1.2, USA). Normally distributed data were expressed as mean ± standard deviation, and between-group comparisons were conducted using independent sample t-tests. Non-normally distributed data were described using median (interquartile range, IQR) and analyzed using the Kruskal-Wallis H test. Count data were summarized using frequencies, and differences between groups were compared using the chi-square test or Fisher’s exact test. Pearson’s or Spearman’s correlation analyses were conducted for correlation analysis. Univariate logistic regression analysis, random forest analysis and multivariate binary logistic regression analysis were performed to identify independent risk factors for pSS combined with CHD, and a new predictive nomogram was constructed based on these risk factors. The discriminative ability of the model was evaluated using the area under the receiver operating characteristic curve (AUROC) and the C-index; the calibration of the model was assessed using calibration curves and the Hosmer-Lemeshow test; the clinical effectiveness of the model was validated using decision curve analysis (DCA) curves.

## 3 Results

### 3.1 Comparisons between the basic information, clinical characteristics and laboratory data of pSS group and PSS-CHD group

This study included a total of 120 patients with pSS, including 60 patients with pSS-CHD. The basic information, clinical characteristics, and laboratory data of the pSS group and pSS-CHD group are presented in **Table 1**. Patients in the pSS-CHD group had a mean age of 60.80 ± 9.24 years, with males comprising 46.7% of the cohort. We selected pure pSS patients matched for age and sex (p = 0.153; p = 0.584). There was a significant difference in disease duration, with patients in the pSS-CHD group exhibiting a longer duration of illness compared to those in the pSS group. Some patients in both groups had received treatment, including NSAIDs, conventional synthetic disease-modifying antirheumatic drugs (csDMARDs), biological DMARDs (bDMARDs), and/or corticosteroids, but no significant differences were noted in treatment regimens. Notably, the prevalence of hypertension was significantly higher in the pSS-CHD group (63.3%) compared to the pSS group (26.7%), while the prevalence of diabetes in the pSS-CHD group was 16.7%, greater than the 5.0% observed in the pSS group. Laboratory data analysis showed that the red blood cell (RBC) count and lymphocyte percentage (LY%) were lower in the pSS-CHD group compared to the pSS group, while the monocyte count was higher in the pSS-CHD group. Aspartate aminotransferase (AST) and direct bilirubin (DBIL) levels were higher in the pSS-CHD group compared to the pSS group. Blood urea nitrogen (BUN), creatinine (Cr), and uric acid (UA) levels were lower in the pSS group compared to the pSS-CHD group. ESR and CRP were significantly higher in the pSS-CHD group compared to the pSS group. Lipid levels were significantly elevated in the pSS-CHD group, particularly cholesterol (CHOL), triglycerides (TG), and low-density lipoprotein cholesterol (LDL-C), compared to the pSS group. In terms of immunological parameters, IgA levels were significantly lower in the pSS group compared to the pSS-CHD group. No statistical differences were observed in the remaining parameters.

**Table 1:** Clinical characteristics of pSS group and pSS-CHD group.

	pSS(n=60)	pSS-CHD(n=60)	*P*
Demographics			
Age(Years)^a^	58.02 ± 11.81	60.80 ± 9.24	0.153
Male, n(%)^b^	29(48.3%)	32(53.3%)	0.584
Female, n(%)^b^	31(51.7%)	28(46.7%)	
BMI^a^	23.47 ± 3.26	24.11 ± 3.84	0.328
Course of disease (month)^c^	31.50(10.25-58.75)	43.00(19.00-72.25)	0.019*
Traditional risk factors			
Smoking, n(%)^b^	2(3.3%)	6(10%)	0.143
Drinking, n(%) ^b^	1(1.7%)	5(8.3%)	0.094
Hypertension, n(%)^b^	16(26.7%)	38(63.3%)	<0.001***
Diabetes, n(%)^b^	3(5.0%)	10(16.7%)	0.040*
Treatment			
NSAIDs, n (%)^b^	10(16.7%)	9(15.0%)	0.803
csDMARDs, n (%)^b^	14(23.3%)	22(36.7%)	0.111
bDMARDs, n (%)^b^	2(3.3%)	1(1.7%)	0.559
GC, n (%)^b^	11(18.3%)	17(28.3%)	0.195
Laboratory Characteristics			
anti-SSA+, n (%)^b^	50(83.3%)	54(90.0%)	0.283
anti-SSB+, n (%)^b^	19(43.2%)	25(56.8%)	0.256
ESR(mm/h)^c^	12.50(8.00-16.00)	28.00(14.50-50.00)	<0.001***
CRP(mg/L)^c^	1.19(0.80-1.97)	3.90(1.88-16.18)	<0.001***
Complete blood count			
WBC(*10^9^/L)^c^	4.68(3.94-6.73)	5.48(4.39-7.13)	0.114
RBC(*10^12^/L)^c^	4.30(3.93-4.53)	4.01(3.68-4.34)	0.014*
Hb(g/L)^c^	129.00(119.50-135.50)	125.50(111.50-137.50)	0.375
PLT(*10^9^/L)^c^	200.50(150.00-263.50)	202.00(146.00-232.50)	0.443
LY(*10^9^/L)^c^	1.56(1.15-2.16)	1.34(0.99-1.92)	0.158
MONO(*10^9^/L)^c^	0.37(0.29-0.49)	0.45(0.35-0.54)	0.014*
NEUT(*10^9^/L)^c^	2.68(1.83-4.10)	3.39(2.47-4.67)	0.058
LY%^c^	33.95(24.00-40.45)	29.50(16.70-37.70)	0.035*
MONO%^c^	7.20(5.95-9.45)	8.10(6.70-9.60)	0.192
NEUT%^c^	57.05(48.65-67.50)	58.35(52.50-67.55)	0.278
Liver Function Test			
ALT(U/L)^c^	16.70(12.40-21.85)	16.65(12.90-25.10)	0.667
AST(U/L)^c^	21.25(16.40-24.15)	23.60(19.15-28.45)	0.017*
TBIL(μmol/L)^c^	11.15(9.55-14.80)	14.25(9.60-18.85)	0.050
DBIL(μmol/L)^c^	2.20(1.80-2.80)	2.65(1.80-4.25)	0.018*
IBIL(μmol/L)^c^	8.90(7.60-12.10)	11.06(7.88-14.55)	0.094
BUN(mmol/L)^c^	4.40(3.75-5.50)	5.20(4.40-6.35)	0.024*
Cr(μmol/L)^c^	57.00(51.00-60.50)	61.00(54.50-71.50)	0.004**
UA(μmol/L)^c^	257.50(225.00-298.50)	296.00(250.00-364.00)	0.002**
CHOL(mmol/L)^c^	3.90(3.15-4.49)	4.51(4.01-5.62)	<0.001***
TG(mmol/L)^c^	1.16(0.89-1.71)	1.61(1.24-2.32)	<0.001***
HDL-C(mmol/L)^c^	1.17(0.97-1.42)	1.20(0.96-1.37)	0.861
LDL-C(mmol/L)^c^	1.99(1.60-2.39)	2.23(1.97-3.29)	0.001**
Immunoglobulin			
IgG(g/L)^c^	14.86(12.21-18.89)	14.60(12.32-19.02)	0.879
IgA(g/L)^c^	2.77(2.16-3.35)	3.08(2.39-4.27)	0.028*
IgM(g/L)^c^	1.02(0.83-1.43)	1.10(0.72-1.86)	0.856

a Date with mean ± standard deviation.

b Data with number (n)/percentage (%).

C Date with median and 25th and 75th percentiles.

BMI, Body Mass Index; NSAIDs, Nonsteroidal Antiinflammatory Drugs; csDMARDs, Conventional synthetic disease-modifying antirheumatic drugs; bDMARD, biological disease-modifying antirheumatic drug; GC, Glucocorticoid; ESR, Erythrocyte sedimentation rate; CRP, C-reactive protein; WBC, White blood cell; RBC, Red blood cell; Hb, hemoglobin; PLT, Platelet; LY, Lymphocyte; MONO, Monocyte; NEUT, Neutrophils; ALT, Alanine transaminase; AST, Aspartic transaminase; TBIL, Total bilirubin; DBIL, Direct bilirubin; IBIL, Indirect bilirubin; BUN, Blood urea nitrogen; Cr, Serum creatinine; UA, Uric acid; CHOL, Cholesterol; TG, Triglycerides; HDL-C, High density lipoprotein cholesterol; LDL-C, Low-density lipoprotein cholesterol; IgG, Immunoglobulin G; IgA, Immunoglobulin A; IgM, Immunoglobulin M. *P<0.05, **P<0.01, ***P<0.001.

### 3.2 Differences in peripheral blood lymphocyte subsets and CD4+ T cell levels between the pSS and pSS-CHD groups

**Figure 1:**
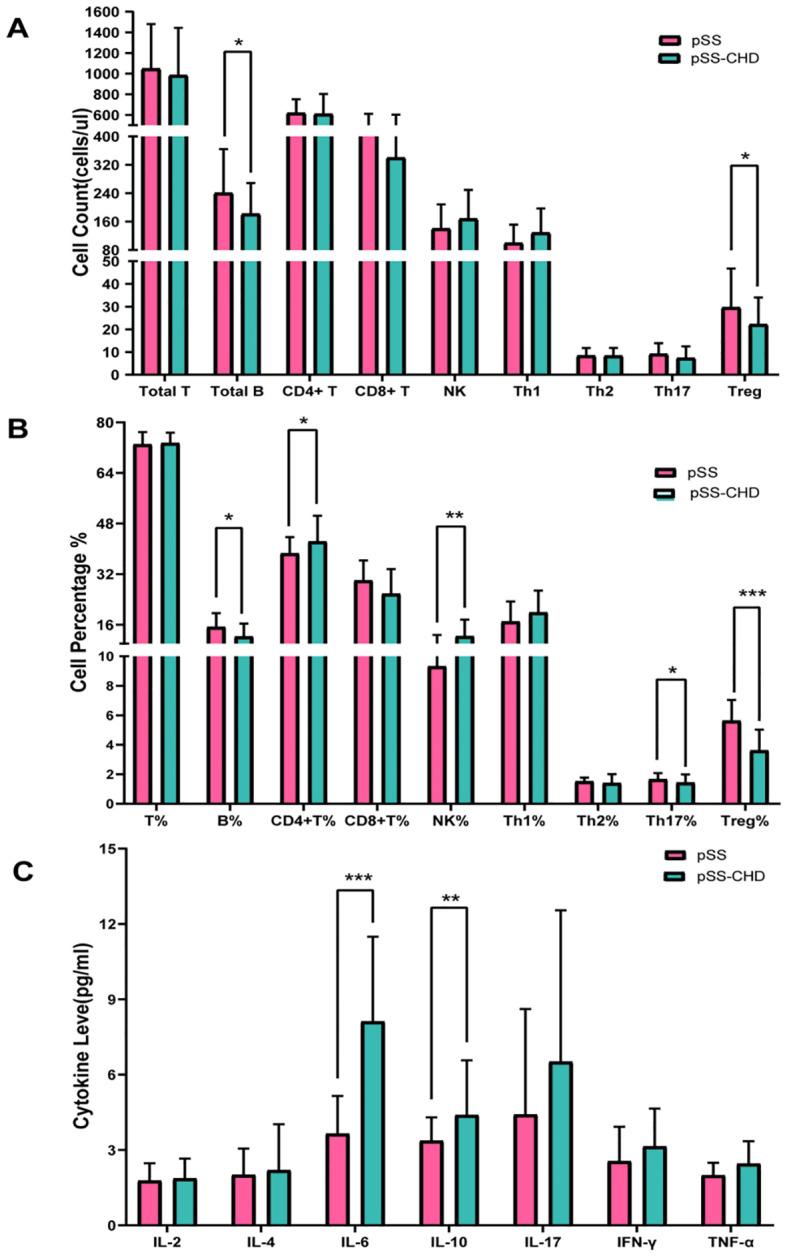
**(A)** Comparison of peripheral blood lymphocyte counts in pSS and pSS-CHD. **(B)** Comparison of the proportion of peripheral blood lymphocyte in pSS and pSS-CHD. **(C)** Comparison of cytokines in pSS and PSS-CHD groups. (*p < 0.05, **p < 0.01, *** p < 0.001).

In this study, the number and percentage of peripheral blood lymphocyte subsets in patients with PSS-CHD and pSS were compared. The results showed that the number and percentage of total B cells in patients with PSS-CHD were lower than those in pSS patients, while the percentage of NK cells, CD4+T cells and CD4+T/CD8+T were significantly higher than those in pSS patients. Further analysis of CD4+T cell subsets showed that the percentage of Th17, the absolute number and percentage of Treg in PSS-CHD group were significantly lower than those in pSS group, while Th1/Treg, Th2/Treg, NKcell/Treg were higher than those in pSS group. There was no statistical difference in other indicators. (**Tables 2**, **3**, **Figures 1A, B**).

**Table 2:** Absolute counts of lymphocyte in the peripheral blood in pSS and pSS-CHD.

	pSS(n=60)	pSS-CHD(n=60)	*P*
Total T(cells/μL)	1046.25(778.66-1479.56)	982.04(741.88-1435.64)	0.510
Total B(cells/μL)	240.33(101.21-357.18)	181.39(82.96-264.24)	0.027*
NK(cells/μL)	139.72(86.49-207.05)	167.64(90.99-248.63)	0.152
CD4+ T(cells/μL)	617.38(375.65-750.90)	608.60(353.75-799.03)	0.834
CD8+ T(cells/μL)	419.61(310.16-606.40)	339.53(245.25-590.23)	0.204
Th1(cells/μL)	99.45(55.99-151.14)	128.33(61.19-196.79)	0.095
Th2(cells/μL)	8.31(5.19-11.50)	8.29(5.32-11.76)	0.642
Th17(cells/μL)	9.05(6.33-13.87)	7.28(4.01-12.37)	0.145
Treg(cells/μL)	29.58(18.71-46.65)	22.11(15.86-33.96)	0.013*

Date with median and 25th and 75th percentiles.

T, T lymphocyte; B, B lymphocyte; NK, Natural killer cell; Th1, T-helper 1 cells; Th2, T-helper 2 cells; Th17, T-helper17 cells; Treg, Regulatory T cells. *P<0.05.

**Table 3:** Proportion of lymphocyte in the peripheral blood in pSS and pSS-CHD.

	pSS(n=60)	pSS-CHD(n=60)	*P*
T%	73.04(66.39-76.89)	73.46(66.12-76.65)	0.830
B%	15.33(10.66-19.61)	12.25(7.57-16.18)	0.027*
NK%	9.32(6.38-12.60)	12.34(9.13-17.58)	0.005**
CD4+ T%	38.59(30.35-43.63)	42.33(34.14-50.29)	0.021*
CD8+ T%	29.98(23.97-36.07)	25.77(21.20-33.58)	0.054
CD4+ T/CD8+ T	1.43(0.89-1.65)	1.75(1.21-2.45)	0.005**
Th1%	17.03(13.28-23.31)	19.91(14.54-26.59)	0.093
Th2%	1.52(1.05-1.77)	1.41(1.05-2.01)	0.939
Th17%	1.67(1.33-2.08)	1.44(0.87-1.97)	0.021*
Treg%	5.64(4.35-6.98)	3.62(3.08-5.01)	<0.001***
Th1/Th2	12.58(9.62-15.15)	13.29(7.61-23.14)	0.268
Th1/Treg	3.34(2.27-5.15)	4.77(3.48-6.47)	0.002**
Th2/Treg	0.30(0.19-0.41)	0.35(0.25-0.51)	0.018*
Th17/Treg	0.33(0.24-0.46)	0.33(0.21-0.52)	0.966
B/Treg	8.64(5.40-11.28)	6.56(4.32-11.96)	0.258
NK/Treg	5.04(3.57-7.58)	6.77(3.58-12.45)	0.016*

Date with median and 25th and 75th percentiles.

T, T lymphocyte; B, B lymphocyte; NK, Natural killer cell; Th1, T-helper 1 cells; Th2, T-helper 2 cells; Th17, T-helper17 cells; Treg, Regulatory T cells. *P<0.05, **P<0.01, ***P<0.001.

### 3.3 Differences in cytokine levels between patients in the pSS and pSS-CHD groups

This study compared the differences in cytokines between the two groups and found that the levels of some cytokines were significantly increased in the pSS-CHD group. Including IL-6 (3.65 vs. 8.12, p < 0.001), IL-10 (3.37 vs. 4.39, p = 0.003), the TNF-α (1.99 vs. 2.45, p = 0.030). These results suggest that abnormalities in many cytokines may be involved in the pathogenesis of disease in pSS-CHD patients (**Table 4**, **Figure 1C**).

**Table 4:** Cytokine levels in the peripheral blood in pSS and pSS-CHD.

	pSS(n=60)	pSS-CHD(n=60)	*P*
IL-2(pg/ml)	1.78(1.26-2.47)	1.87(1.22-2.65)	0.962
IL-4(pg/ml)	2.01(1.32-3.06)	2.19(1.36-3.93)	0.400
IL-6(pg/ml)	3.65(2.90-5.08)	8.12(4.93-11.46)	<0.001***
IL-10(pg/ml)	3.37(2.61-4.27)	4.39(3.02-6.48)	0.003**
IL-17(pg/ml)	4.41(1.48-8.61)	6.51(2.12-12.51)	0.141
IFN-γ(pg/ml)	2.55(1.87-3.92)	3.14(2.14-4.62)	0.079
TNF-α(pg/ml)	1.99(1.36-2.49)	2.45(1.77-3.34)	0.030*

Date with median and 25th and 75th percentiles.

IL-2, Interleukin-2; IL-4, Interleukin-4; IL-6, Interleukin-6; IL-10, Interleukin-10; IL-17, Interleukin-17; INF-γ, Interferon-γ; TNF-α, Tumor necrosis factor-α. *P<0.05, **P<0.01, ***P<0.001.

### 3.4 Development of the nomogram for prediction of CHD in pSS

**Figure 2:**
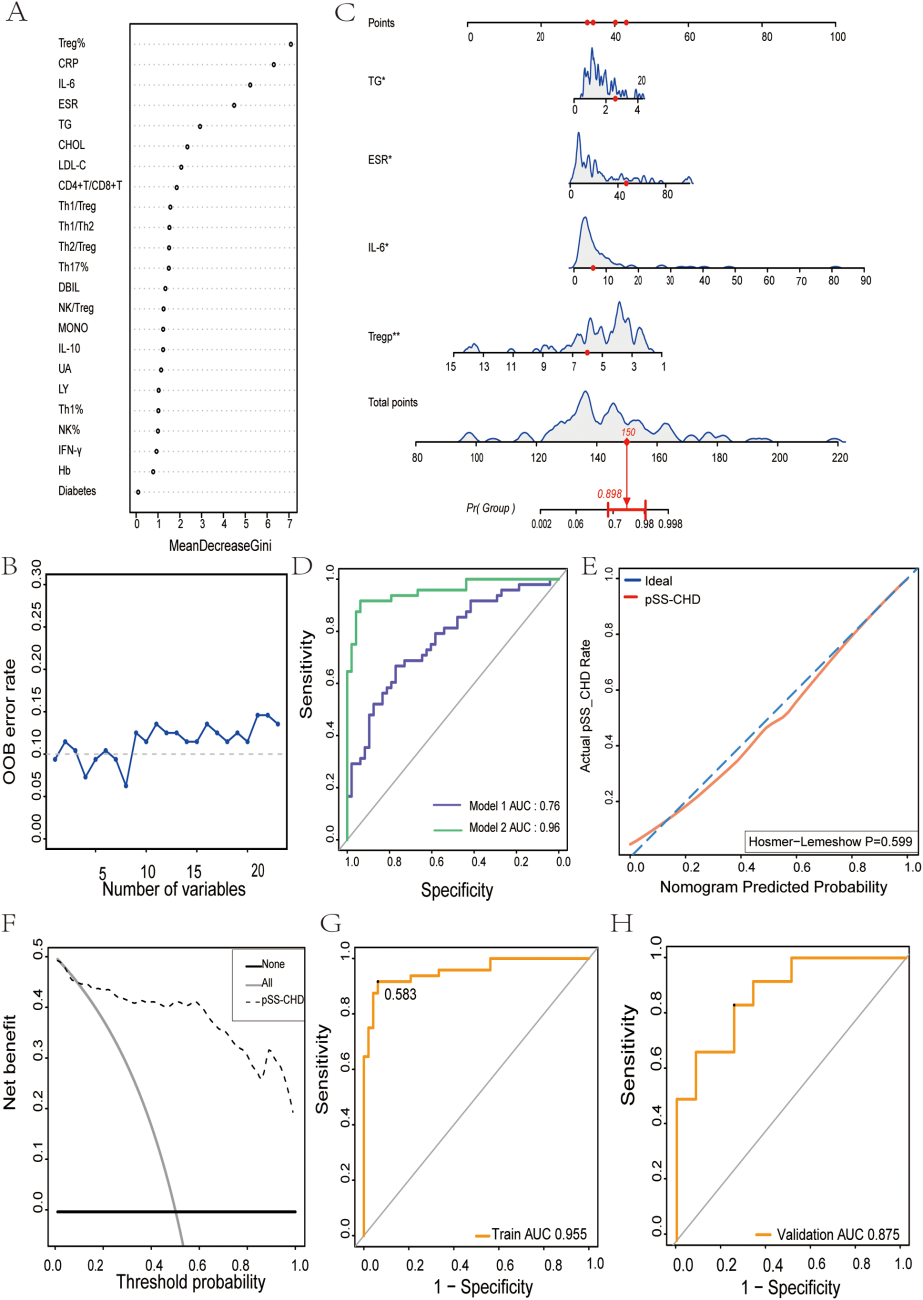
The nomogram for predicting the development and evaluation of CHD in patients with pSS. **(A)** Screening of important predictors of CHD risk in pSS patients. **(B)** The relationship between the number of CHD risk predictors and the OOB error rate in pSS patients. **(C)** Nomogram for predicting CHD risk in pSS patients. **(D)** Receiver Operating characteristics (ROC) curve for prediction of CHD risk in pSS patients. Model 1: Risk factors involved in nomogram include ESR and TG. Model 2: Risk factors involved in nomogram include ESR, TG, IL-6, and Treg%. **(E)** Calibration curve for CHD risk prediction in pSS patients. **(F)** Decision analysis curve for CHD risk prediction in pSS patients. **(G)** The nomogram described by receiver operating characteristic (ROC) curve was used to determine the CHD risk of the model population. **(H)** Nomogram of receiver operating characteristics (ROC) curves to determine CHD risk in the validated population. *p<0.05. **p<0.01.

A total of 120 pSS patients were randomly assigned to a modeling group (n = 96) and a validation group (n = 24) at a ratio of 8:2 by using a random number table. There were no significant differences in clinical data, laboratory data and peripheral blood lymphocyte subsets between the two groups (P>0.05). Firstly, univariate Logistic regression was used to screen the risk factors of pSS patients with CHD. There were significant differences in clinical indicators between the two groups (P<0.05), as shown in **Table 5**. Next, variables with statistical differences screened by univariate Logistics regression were included in a random forest analysis to explore the importance of variables and evaluate the predictive effect of different variables in pSS patients with CHD. The importance of variables was ranked according to the average decrease of Gini value (MDG) in the random forest model, and stepwise random forest regression analysis was performed from high to low according to the ranking result. The results showed that when the number of variables was 8, the out-of-bag error estimation rate was the lowest (OBB), and the variable was ranked according to the importance of the variable. The top 8 variables Trep%, CRP, IL-6, ESR, TG, CHOL, LDL-C, CD4+T/CD8+T were included in the multivariate logistic stepwise regression analysis. The results showed that in addition to ESR, TG and other traditional risk factors, IL-6 and Trep% may also be independent risk factors for CHD in pSS patients (**Table 6**, **Figures 2A, B**). At the same time, the above four independent factors were included in the prediction model, and a nomogram prediction model for pSS patients with CHD was established (**Figure 2C**). Considering that IL-6 and Trep% are not well-known traditional risk factors for CHD, IL-6 and Trep% were removed from the nomogram prediction model, and a new prediction model 1 was established, and the test efficacy of the two models was compared.

**Table 5:** Univariate logistic regression analyses for factors associated with the presence of CHD in pSS patients.

Variables	OR	95%CI	*P*
Demographics			
Age	1.03	0.99-1.07	0.127
Sex	1.18	0.53-2.63	0.683
BMI	0.99	0.96-1.02	0.530
Course of disease	1.013	1.00-1.03	0.041*
Traditional risk factors			
Smoking	3.29	0.63-17.18	0.159
Drinking	5.47	0.61-48.66	0.128
Hypertension	5.38	2.24-12.92	<0.001***
Diabetes	3.95	1.01-15.39	0.048*
Treatment			
NSAIDs	0.88	0.33-2.35	0.803
csDMARDs	1.90	0.86-4.22	0.113
bDMARDs	0.49	0.04-5.57	0.566
GC	1.76	074-4.17	0.198
Laboratory Characteristics			
anti-SSA+	1.80	0.61-5.32	0.287
anti-SSB+	1.54	0.73-3.26	0.257
ESR	1.07	1.03-1.11	<0.001***
CRP	1.99	1.40-2.84	<0.001***
Complete blood count			
WBC	1.19	0.99-1.43	0.064
RBC	0.50	0.22-1.16	0.106
HGB	0.99	0.96-1.01	0.275
PLT	1.00	0.99-1.01	0.955
LY	1.02	0.94-1.12	0.586
LY%	0.96	0.92-0.99	0.015*
MONO	1.19	0.98-1.45	0.090
MONO%	1.01	0.89-1.14	0.862
NEUT	1.28	1.00-1.64	0.050
NEUT%	1.02	0.99-1.05	0.231
Liver Function Test			
ALT	1.03	0.99-1.08	0.097
AST	1.05	1.00-1.11	0.068
TBIL	1.07	1.00-1.15	0.065
DBIL	1.53	1.09-2.14	0.014*
IBIL	1.07	0.98-1.17	0.119
Renal Function Test			
BUN	1.21	0.96-1.53	0.098
Cr	1.03	1.00-1.06	0.073
UA	1.01	1.00-1.02	0.005**
Blood Lipid Test			
CHOL	2.31	1.45-3.67	<0.001***
TG	2.34	1.3-4.21	0.005**
HDL-C	0.90	0.30-2.72	0.855
LDL-C	2.46	1.38-4.38	0.002**
Immunoglobulin			
IgG	1.00	0.97-1.04	0.857
IgA	1.32	0.94-1.86	0.108
IgM	1.21	0.86-1.71	0.281
lymphocyte			
Total T	1.00	1.00-1.00	0.776
T%	0.98	0.95-1.02	0.407
Total B	1.00	1.00-1.00	0.109
B%	0.98	0.94-1.02	0.340
NK	1.00	1.00-1.01	0.058
NK%	1.08	1.01-1.15	0.020*
CD4+T	1.00	1.00-1.00	0.366
CD4+ T%	1.00	1.00-1.00	0.557
CD8+ T	1.00	1.00-1.00	0.811
CD8+ T%	0.96	0.93-1.00	0.065
CD4+ T/CD8+ T	2.12	1.24-3.65	0.006**
Th1	1.00	1.00-1.00	0.318
Th1%	1.05	1.00-1.1	0.037*
Th2	1.02	0.96-1.09	0.448
Th2%	1.19	0.60-2.38	0.617
Th17	1.00	0.95-1.05	0.985
Th17%	0.44	0.22-0.85	0.015*
Treg	0.98	0.95-1.00	0.053
Treg%	0.45	0.32-0.65	<0.001***
Th1/Th2	1.05	1.00-1.11	0.034
Th1/Treg	1.31	1.09-1.58	0.004**
Th2/Treg	12.89	1.41-118.11	0.020*
Th17/Treg	3.15	0.46-21.42	0.241
B/Treg	0.99	0.93-1.05	0.692
NK/Treg	1.11	1.01-1.22	0.026*
Cytokine			
IL-2	1.01	0.72-1.40	0.973
IL-4	1.30	0.99-1.69	0.059
IL-6	1.46	1.20-1.77	<0.001***
IL-10	1.33	1.05-1.70	0.020*
IL-17	1.02	0.98-1.07	0.255
IFN-γ	1.34	1.04-1.73	0.023*
TNF-α	1.35	0.98-1.85	0.064

Date with median and 25th and 75th percentiles.

BMI, Body Mass Index; ESR, Erythrocyte sedimentation rate; CRP, C-reactive protein; WBC, White blood cell; RBC, Red blood cell; Hb, Hemoglobin; PLT, Platelet; LY, Lymphocyte; MONO, Monocyte; NEUT, Neutrophils; ALT, Alanine transaminase; AST, Aspartic transaminase; TBIL, Total bilirubin; DBIL, Direct bilirubin; IBIL, Indirect bilirubin; BUN, Blood urea nitrogen; Cr, Serum creatinine; UA, Uric acid; CHOL, Cholesterol; TG, Triglycerides; HDL-C, High density lipoprotein cholesterol; LDL-C, Low-density lipoprotein cholesterol; IgG, Immunoglobulin G; IgA, Immunoglobulin A; IgM, Immunoglobulin M; T, T lymphocyte; B, B lymphocyte; NK, Natural killer cell; Th1, T-helper 1 cells; Th2, T-helper 2 cells; Th17, T-helper17 cells; Treg, Regulatory T cells; IL-2, Interleukin-2; IL-4, Interleukin-4; IL-6, Interleukin-6; IL-10, Interleukin-10; IL-17, Interleukin-17; INF-γ, Interferon-γ; TNF-α, Tumor necrosis factor-α; OR, Odds ratio; 95%CI, 95% confidence interval. *P<0.05, **P<0.01,***P<0.001.

**Table 6:** multivariate logistic regression analyses for factors associated with the presence of CHD in pSS patients.

Variables	OR	95%CI	*P*
ESR	1.10	1.03-1.22	0.019*
CRP	1.72	1.11-3.71	0.094
CHOL	2.05	0.71-8.51	0.058
TG	3.67	0.96-19.16	0.041*
LDL-C	2.05	0.27-17.9	0.490
CD4+ T/CD8+ T	1.25	0.54-4.3	0.680
Treg%	0.25	0.08-0.54	0.004**
IL-6	1.29	1.00-1.68	0.048*

Date with median and 25th and 75th percentiles.

ESR, erythrocyte sedimentation rate; CRP, C-reactive protein; CHOL, Cholesterol; TG, Triglycerides; LDL-C, Low-density lipoprotein cholesterol; Treg, Regulatory T cells; IL-6: Interleukin-6; OR, Odds ratio; 95%CI, 95% confidence interval. *P<0.05, **P<0.01.

### 3.5 Validation of the nomogram

Through comparing the two models, we found that the AUC of Model 1 was 0.76, while the AUC of Model 2 was 0.96. Model 2 exhibited superior discriminative ability over Model 1 (**Figure 2D**). This suggests that peripheral blood Trep% and IL-6 may play important roles in predicting the onset of CHD in pSS patients.

To further validate the discriminative ability of the models, we plotted ROC curves for both groups of patients. The AUC for the model population was 0.955 (**Figure 2G**), while for the validation population, it was 0.875 (**Figure 2H**). The AUCs in both populations were > 0.75, indicating good discriminative ability of the models.

To assess the calibration of the models, calibration curves were plotted, showing that the closer the predicted curve was to the observed curve, the better the calibration ability of the model. Furthermore, the Hosmer-Lemeshow test was conducted, yielding a p-value of 0.599 (P>0.05) (**Figure 2E**).

Additionally, clinical decision curves (DCA) were plotted, revealing that the cut-off value (58.3%) obtained from the ROC analysis fell within the threshold probability range of the DCA curve. Further analysis showed that when the threshold probability for diagnosing CHD in pSS was set at 58.3%, 45 out of 100 pSS patients at risk of CHD could benefit from the model without harming others (**Figure 2F**).

### 3.6 Correlation analysis of peripheral blood Treg% and IL-6with dyslipidemia in patients with primary Sjögren’s syndrome

**Figure 3:**
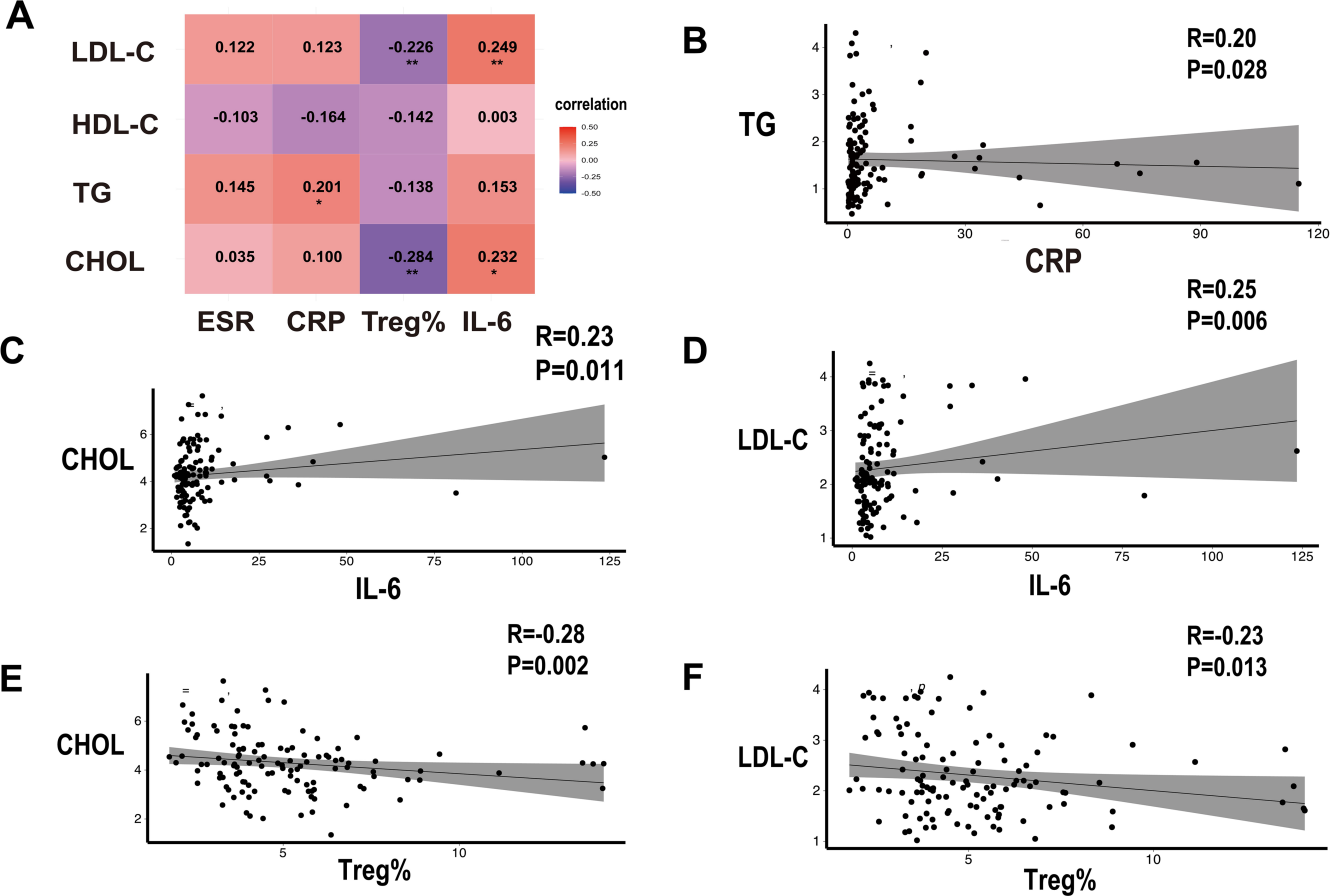
**(A)** Heatmap illustrating the correlation between ESR, CRP, Treg%, IL-6, and lipid profile parameters in patients with primary Sjögren’s syndrome (pSS). **(B)** Correlation between CRP and triglycerides (TG) in pSS patients. **(C, D)** Correlation of IL-6 with cholesterol (CHOL) and low-density lipoprotein cholesterol (LDL-C) in pSS patients. **(E, F)** Correlation of Treg% with CHOL and LDL-C in pSS patients. *p < 0.05, **p < 0.01.

Dyslipidemia is a significant traditional risk factor for CHD. In this study, we found that the lipid levels (including CHOL, TG, and LDL-C) in the pSS-CHD group were significantly higher than those in the pure pSS group. Further analysis of the lipid profile in patients with pSS revealed correlations with inflammatory markers (such as ESR and CRP), Treg%, and IL-6. The results indicated a negative correlation between peripheral blood Treg% and both CHOL and LDL-c, whereas IL-6 showed a positive correlation with these lipid parameters. Additionally, TG were positively correlated with CRP (see **Figures 3A–F**).

## 4 Discussion

CHD is one of the leading causes of mortality worldwide, with patients suffering from pSS exhibiting an elevated risk for CHD. This heightened risk may arise from the interplay between immune-inflammatory responses and traditional risk factors. Historically, research on CHD risk in pSS patients has predominantly focused on traditional risk factors or inflammatory biomarkers (9), with insufficient exploration of the levels of immune cells and cytokines, as well as their interrelationships. While individualized predictive models for cardiovascular disease risk have been developed in chronic autoimmune conditions such as systemic lupus erythematosus and rheumatoid arthritis (34, 35), predictive models specifically for pSS patients with CHD remain limited. To enhance the identification of pSS patients at risk for CHD, this study aims to develop and validate a nomogram for assessing CHD risk in this population. Notably, this research represents the first instance of constructing a predictive model for CHD risk in pSS patients utilizing electronic medical record (EMR) data.

To identify key distinguishing factors between patients with pSS and those with pSS combined with CHD, we initially performed univariate logistic regression analysis to compare demographic characteristics, laboratory parameters, and immunological markers. We then applied a random forest algorithm to rank the importance of these key indicators. Subsequent multivariate logistic regression analysis revealed that TG, ESR, IL-6, and Treg% are independent risk factors for CHD. Based on these factors, we constructed a nomogram, which integrates multiple predictive indicators into a visually intuitive format, allowing for a clear representation of the relationships among independent risk factors in the predictive model. Each influencing factor is assigned a weight based on its contribution to the outcome variable, and the total score is calculated by summing these weighted scores. For instance, when the total score reaches 150, the corresponding risk of developing CHD is 0.898.

To further validate the role of immunological markers in the predictive model, we excluded IL-6 and Treg% during model construction and evaluated the predictive performance of the two models using receiver operating characteristic (ROC) curves. The results indicated a decline in discriminative ability for the new model, underscoring the critical roles of IL-6 and Treg% in predicting CHD. The optimal cutoff point was determined based on the Youden index (Youden index = sensitivity + specificity - 1), with the maximum Youden index corresponding to the best threshold value. In this study, the optimal cutoff was found to be 58.3%. When used as a threshold for decision curve analysis (DCA), the model demonstrated a high clinical net benefit, suggesting that interventions should be considered when the predicted risk exceeds 58.3%. In summary, these results indicate that the user-friendly nomogram effectively predicts CHD risk in pSS patients, enabling clinicians to quantify the risk and implement early intervention strategies in high-risk populations, thereby optimizing treatment approaches. Additionally, the model can serve as an educational tool for patients, enhancing their understanding of the importance of controlling risk factors and improving treatment adherence.

In this study, the changes in IL-6 levels and Treg percentages play a critical role in the progression of CHD in patients with pSS. IL-6 is a multifunctional pro-inflammatory cytokine that is pivotal in regulating immune responses and inflammatory processes (36, 37). In pSS patients, a chronic low-grade inflammatory state is often accompanied by a significant increase in IL-6 levels. This chronic inflammation can impair endothelial cell function and induce atherosclerosis (15, 38). IL-6 exacerbates the inflammatory response in arterial walls by influencing lipid metabolism, leading to increased levels of CHOL and TG, promoting foam cell formation, and facilitating lipid deposition in the vascular wall (39, 40). Additionally, IL-6 enhances B cell activation and antibody production, resulting in the formation of immune complexes. These immune complexes can deposit on the vascular endothelium, inciting local inflammatory responses and damaging vascular structures, thereby propelling the progression of atherosclerosis (41). Recent studies have shown that IL-6 promotes the proliferation and migration of vascular smooth muscle cells (VSMCs), leading to intimal thickening and vascular narrowing, significantly increasing the risk of CHD. The underlying mechanisms involve IL-6 triggering signaling pathways such as p38 MAPK and ERK1/2, which enhance VSMC activity and exacerbate the atherosclerotic process. This cytokine-driven inflammation is a key factor in the progression of atherosclerosis, which is fundamental to the development of CHD (42).

Treg cells, a subset of CD4+ T lymphocytes, primarily protect the body from autoimmune attacks by suppressing inflammatory responses and maintaining immune tolerance (43). In patients with pSS and CHD, the number and proportion of Treg cells are significantly reduced. This decrease may lead to an imbalance in the immune system’s regulation of effector T cells, promoting uncontrolled inflammatory responses and releasing more pro-inflammatory cytokines such as IL-6 and TNF-α, thereby exacerbating endothelial injury (41). Recent studies indicate that Treg cells exert anti-inflammatory effects by regulating the secretion of cytokines like IL-10 and TGF-β, which play crucial roles in immune modulation and inflammation control. The reduction in Treg% can impair this regulatory function, leading to heightened inflammatory responses (44). Specifically, diminished Treg activity may result in the loss of inhibition over effector T cells and other pro-inflammatory cytokines (45), potentially further exacerbating inflammation associated with CHD in pSS patients. Additionally, in the context of chronic inflammation, a deficiency of Treg cells may alter vascular smooth muscle cell function, impacting the vascular remodeling process, promoting atherosclerosis and plaque formation, and increasing the risk of CHD (46).

Studies have also shown that patients with pSS and CHD often exhibit dyslipidemia, characterized by elevated levels of total cholesterol, triglycerides, and LDL-C, alongside reduced levels of HDL-C. The interaction between the immune and metabolic systems is reflected in the negative correlation between Treg% and CHOL and LDL-C, while IL-6 shows a positive correlation with these lipid parameters. These findings further underscore the significance of immune inflammatory responses in the risk of developing CHD in pSS patients. Moreover, research indicates that abnormal lipid levels may influence the metabolic pathways and functions of Treg cells, such as the regulation of the mTOR signaling pathway, thereby weakening their immunosuppressive capacity and exacerbating inflammation (47). Future studies should explore the specific mechanisms by which Treg cells are involved in metabolic abnormalities and the progression of atherosclerosis, to elucidate how treatment strategies can be optimized to improve the prognosis of pSS patients with CHD.

In fact, our study has several limitations. First, it was conducted as a single-center investigation, resulting in a limited sample size, which raises concerns about potential overfitting and may affect the robustness of the predictive nomogram. Future research should aim to externally validate the model using multicenter patient data to enhance its reliability. Second, this retrospective study based on collected clinical data is subject to inevitable limitations, such as incomplete clinical records leading to a relatively small number of enrolled cases. Additionally, we were unable to assess the efficacy of statin therapy on hyperlipidemia, and there may be delays in diagnosing CHD in pSS-CHD patients, even when the condition existed prior to the onset of pSS. Prospective studies are necessary to mitigate these confounding factors. Third, our analysis relied entirely on clinical data; incorporating additional biomarkers could further improve the diagnostic accuracy of the model. Fourth, future efforts should focus on designing larger, more diverse randomized clinical trials or cohort studies to validate the model’s predictive capability for CHD in pSS patients, thereby increasing its generalizability. Moreover, it is important to evaluate the model’s predictive ability concerning treatment interventions. Clinical predictive models can guide healthcare professionals in decision-making, ultimately enhancing patient outcomes and societal benefits. Additionally, these models provide an objective assessment of risks for both patients and healthcare providers, facilitating subjective interpretations and intuitions, as well as informing guidelines (48).

In summary, this study aims to investigate the biological significance of IL-6 and Treg% in patients with pSS and to evaluate their role in the CHD risk prediction model. The results indicate that both IL-6 and Treg% serve not only as potential biomarkers but also as critical components in the construction of the CHD risk prediction model. By integrating these immunological indicators with traditional risk factors, the developed nomogram can provide accurate individualized risk assessments for pSS patients with CHD, guiding early clinical interventions to enhance patient quality of life. This prediction model demonstrates good discriminative ability, calibration, and clinical validity, suggesting its potential for significant clinical guidance in the future. Furthermore, the correlation of Treg% and IL-6 with dyslipidemia in pSS patients suggests that these markers may play a key role in the development of CHD and can serve as potential biomarkers for assessing inflammatory status in pSS patients. Future research will further explore the specific molecular mechanisms by which Treg% and IL-6 contributes to the pathogenesis of CHD risk in pSS, enhancing our understanding of their roles in this context.

## Data Availability

The original contributions presented in the study are included in the article/supplementary material. Further inquiries can be directed to the corresponding authors.
